# Orofacial clefts lead to increased pro-inflammatory cytokine levels on neonatal oral mucosa

**DOI:** 10.3389/fimmu.2022.1044249

**Published:** 2022-11-16

**Authors:** Corinna L. Seidel, Elena Percivalle, Marco Tschaftari, Matthias Weider, Karin Strobel, Ines Willershausen, Christoph Unertl, Helga M. Schmetzer, Manuel Weber, Michael Schneider, Benjamin Frey, Udo S. Gaipl, Matthias W. Beckmann, Lina Gölz

**Affiliations:** ^1^ Department of Orthodontics and Orofacial Orthopedics, Universitätsklinikum Erlangen, Friedrich-Alexander-Universität (FAU) Erlangen-Nürnberg, Erlangen, Germany; ^2^ Med III, University Hospital of Munich, Workgroup: Immune modulation, Munich, Germany; ^3^ Department of Oral and Cranio-Maxillofacial Surgery, Friedrich-Alexander-Universität (FAU) Erlangen-Nürnberg, Erlangen, Germany; ^4^ Department of Gynecology and Obstetrics, Comprehensive Cancer Center (CCC) Erlangen-EMN, Universitätsklinikum Erlangen, Friedrich-Alexander-Universität (FAU) Erlangen-Nürnberg, Erlangen, Germany; ^5^ Translational Radiobiology, Department of Radiation Oncology, Universitätsklinikum Erlangen and Friedrich-Alexander-Universität (FAU) Erlangen-Nürnberg, Erlangen, Germany; ^6^ Medical Immunology Campus Erlangen, FAU Erlangen-Nürnberg, Erlangen, Germany

**Keywords:** orofacial clefts, cleft lip and palate, neonates, cytokines, oral, oral inflammation, mucosal immunity, mucosal homeostasis

## Abstract

Orofacial clefts (OFC) are frequent congenital malformations characterized by insufficient separation of oral and nasal cavities and require presurgical infant orthopedics and surgical interventions within the first year of life. Wound healing disorders and higher prevalence of gingivitis and plaque levels are well-known challenges in treatment of children with OFC. However, oral inflammatory mediators were not investigated after birth using non-invasive sampling methods so far. In order to investigate the impact of OFC on oral cytokine levels, we collected tongue smear samples from 15 neonates with OFC and 17 control neonates at two time points (T), T0 at first consultation after birth, and T1, 4 to 5 weeks later. The samples were analyzed using multiplex immunoassay. Overall, we found significantly increased cytokine levels (TNF, IL-1β/-2/-6/-8/-10) in tongue smear samples from neonates with OFC compared to controls, especially at T0. The increase was even more pronounced in neonates with a higher cleft severity. Further, we detected a significant positive correlation between cleft severity score and distinct pro-inflammatory mediators (GM-CSF, IL-1β, IL-6, IL-8) at T0. Further, we found that breast-milk (bottle) feeding was associated with lower levels of pro-inflammatory cytokines (IL-6/-8) in neonates with OFC compared to formula-fed neonates. Our study demonstrated that neonates with OFC, especially with high cleft severity, are characterized by markedly increased inflammatory mediators in tongue smear samples within the first weeks of life potentially presenting a risk for oral inflammatory diseases. Therefore, an inflammatory monitoring of neonates with (severe) OFC and the encouragement of mother to breast-milk (bottle) feed might be advisable after birth and/or prior to cleft surgery.

## 1 Introduction

With an average occurrence of 1 in 700 newborns worldwide, orofacial clefts (OFC) are considered one of the most frequent malformations of craniofacial development (1, 2). OFCs develop between the 5^th^ and the 12^th^ week of embryogenesis due to disturbances in the fusion of the medial/lateral nasal and maxillary processes (3). These defective processes were associated with genetic predisposition (4), e.g., candidate loci 8q24, 1p22 and 10q25 (5, 6), or environmental factors, e.g., maternal smoking, folate deficiency and excessive alcohol consumption (7–9). OFCs present phenotypic variability and severity (10), e.g. unilateral cleft lip and palate (UCLP), bilateral cleft lip and palate (BCLP), isolated cleft palate (CPo) and isolated cleft lip (CLo) (11), leading to altered orofacial anatomical features and functional challenges. Within the first year of life, neonates with OFC require presurgical infant orthopaedics (PSIO), which aims to support preoperative growth of the palatal segments, normalize feeding and reduce asymmetries using palate plates (12, 13) before surgical lip and/or palate repair (12, 13). Even though, feeding difficulties like chronic aspiration and choking can pose a challenge and impede breast-feeding (14, 15) and, therefore, feeding alternatives like Haberman feeders are recommended and applied (16, 17).

In addition to these functional challenges, it is known that infants and adolescents suffer from aggravated oral health compared to controls, e.g., increased plaque levels, enhanced gingival inflammation and deeper periodontal pockets (18). Further, it was recently shown that neonates with OFC suffer from oral dysbiosis with increased levels of potentially pathogenic bacteria, e.g., *Enterobacteriaceae (Citrobacter, Enterobacter, Escherichia-Shigella, Klebsiella), Enterococcus, Bifidobacterium, Corynebacterium, Lactocaseibacillus, Staphylococcus, Acinetobacter and Lawsonella* (19). In infants and children with OFC, pre- and post-operative oral inflammation pose a main risk for wound healing disorders and failure of surgery (20), yet, preventive strategies, e.g. antibiotics prior to cleft lip surgery, did not achieve the desired results (21). Besides, early life inflammation was shown to increase the risk for other inflammation-associated diseases, e.g. autoimmunity (22). Hence, further knowledge about inflammatory processes associated with clefting are necessary, especially in neonates within the first year of life before surgical cleft closure. However, for investigation of oral local inflammation within the first year of life, solely an invasive method was used for investigation of cytokine levels so far: collection of lip tissue from infants with OFC during cleft surgery (3 to 18 months of age) (23, 24). Interestingly, the authors found positive correlations between certain pro-inflammatory cytokines in lip tissue from neonates with OFC (without a control group) (23). Further,overall higher concentrations of cytokines were found in lip tissue from neonates with OFC compared to a non-age-matched and site-matched control group (tissue from extraction site of adolescents) (24). Since the invasive collection of lip tissue (23, 24) is ethically unacceptable in neonates without OFC, non-invasive methods for oral sample collection in neonates are necessary to ensure an age- and site-matched control group. It was recently shown that non-invasive sampling methods were suitable for cytokine and microbiota analyses in oral niches from adults (smear samples from tongue, hard palate, cheek and sublingual area, spitting method for collection of saliva, plaque sampling and collection of gingival crevicular fluid using paper strips) (25) and non-invasive collection of tongue and smear samples for microbiota analyses in neonates with and without OFC (19). Since smear samples from the cheek presented lowest cytokine concentrations in adults (high saliva flow rate: small salivary grands in the cheek) (25) and since no significant microbial differences were seen between niche tongue and cheek (19), non-invasive sampling of tongue smear samples might be the most suitable representative of the oral cavity for cytokine analyses in neonates.

Therefore, the aim of this study was to prove that a non-invasive collection methodology using tongue smear samples from neonates for investigation of cytokines is applicable. We aimed to analyse distinct cytokines (GM-CSF, INF-γ, TNF, IL-1β/-2/-4/-6/-8/-10), that were shown to have anti- or pro-inflammatory functions in wound healing processes, inflammatory pathways and mucosal immunity (**Table 1**), in neonates with OFC compared to neonates without OFC at first consultation after birth and 4 to 5 weeks later. Moreover, the impact of cleft phenotype and severity on cytokine levels were determined. The overarching goal was to increase knowledge about inflammatory changes within the first weeks of life and to identify neonates at risk for oral inflammation and wound healing disorders.

**Table 1:** Measured cytokines and their main functions in wound healing, inflammatory pathways and in mucosal immunity.

Cytokine abbreviation	Source	Functions in wound healing	Functions in inflammatory pathways	Functions on mucosal immunity	References
‘pro-inflammatory’ cytokines					
Granulocyte-macrophage colony-stimulating factor (GM-CSF)	Macro-phages T cells	GM-CSF activated phagocytes cause tissue damage (26)	GM-CSF upregulates CCL17 pathway in inflammation (27) GM-CSF stimulates TNF production (28)	Role in inflammatory signaling and dendritic cell recruitment into mucosa (29)	(26–29)
Interferon gamma (INF-γ)	B/NK/NKT/T cells APCs	activation of antimicrobial and antiviral pathways (30); increase of inflammation-induced tissue damage (31)	leukocyte adhesion, differentiation of immune cells, stimulation of macrophages (30, 32); negatively regulated by IL-4/-10 (30)	weakening of epithelial integrity (migration of bacteria through altered barrier) (30, 31)	(30, 31) (33)
Tumor necrosis factor (TNF)	Macrophages, T cells, NK cells, mast cells	high levels of TNF were associated with surgical site infection (34); TNF inhibitor treatment slightly reduced surgical site infection (35, 36)	Primary pro-inflammatory cytokine (37): vasodilatation, oedema formation, leukocyte adhesion (28); cross-regulation between IL-8, IL-1ß and TNF (37, 38); stimulated by GM-CSF (28)	Stimulation of IL-8 production by mucosal neonatal epithelial cells (39); high levels were associated with worsening of the mucosal epithelial barrier function (40, 41)	(28, 34–36, 38, 40, 42) (43) (37, 39)
Interleukin-1β (IL-1β)	Monocytes macrophages	Primary host defense, response to injury, enhancement of tissue damage in injury-associated mechanisms (44)	Primary pro-inflammatory reactions by the innate immune system, activation of IL-8 (37)	Stimulation of IL-8 production by mucosal neonatal epithelial cells (39); capable to compromise mucosal barrier function (41)	(37, 39, 41, 44)
Interleukin-6 (IL-6)	T cells, macrophages, mast cells	promoting migration of immune cells to the damaged site/wound (45); increased levels were shown to alter tissue integrity (41)	high levels (in plasma) were associated with severity of infectious neonatal diseases (33); produced after IL-1 β, TNF and INF-γ stimulation (32)	capable to compromise mucosal barrier function (41)	(32, 41, 46) (45) (33)
Interleukin-8 (IL-8)	Phagocytes mesenchymal cells mast cells	promotion of tissue destruction (neutrophil accumulation and granules release) (47, 48); reduced IL-8 production associated with scarless wound healing (49)	Secondary pro-inflammatory cytokine in inflammatory reactions by the innate immune system after IL-1β trigger and TNF stimulus (37, 38)	produced under the stimulus of TNF and IL-1β by neonatal nasal mucosa epithelial cells (39); capable to compromise mucosal barrier function (41)	(32, 38, 41, 47–49) (26) (39)
‘pro- and anti-inflammatory’ cytokines					
Interleukin-2 (IL-2)	CD4^+^ T cells	*anti-inflammatory*: treatment with IL-2 promotes tissue integrity, defense, tolerance and strengthens the wound (43, 45, 50)	important regulator in communication of innate and adaptive immunity; activation of T/B/NK cells (32); stimulation of CD8^+^ cytotoxicity (32);	regulation of oral mucosal inflammation (activation of NF-ĸB pathway) (51)	(32, 43, 45, 50, 51)
Interleukin-10 (IL-10)	CD4^+^ T cells B cells monocytes dendritic cells	*anti-inflammatory*: controlling the extend and duration of inflammation in wound healing (major suppressor of immune responses) (52, 53)	*pro-inflammatory*: in a comprised micro-environment (54), upregulated during inflammation when other pro-inflammatory cytokines, e.g., TNF and IL-6, increase (33) IL-10 inhibits the production of IL-1β and TNF (32) high levels (in plasma) were associated with severity of infectious neonatal diseases (33)	promoting oral tolerance (55) anti-inflammatory in mucosal inflammation (down regulation of immune responses to pathogens/microbiota) (52) upregulated during inflammation when other pro-inflammatory cytokines increase, e.g. IL-2, INF-γ in gingival crevicular fluid (56, 57)	(32, 52, 54, 55) (58) (33, 54)
‘anti-inflammatory’ cytokine					
Interleukin-4 (IL-4)	Mast cells, CD4^+^ T cells, Baso-phils, Eosino-phils	important role in wound healing (activation of fibroblasts, keratinocytes, neoangionesis and reepithelization) (46) application of IL-4 accelerates wound healing (59)	decreased levels of IL-4 were correlated to progression of inflammatory diseases (60); antagonistic effects in inflammatory diseases: IL-4 inhibits TNF and IL-1 β production (61)	mucosal wound healing was associated with increased IL-4 levels (62); Anti-inflammatory and immunoregulatory functions in mucosal immune reactivity (60)	(32, 46, 59–62)

APC, antigen presenting cell; NK cell, natural killer cell; NKT cell, natural killer T cell; CD, cluster of differentiation.

## 2 Material and methods

### 2.1 Study design

This study was designed as a prospective, exploratory observational clinical trial and has been approved by the local ethics committee of the Friedrich-Alexander-University Erlangen-Nürnberg (Krankenhausstraße 12, 91054 Erlangen, Vote number: 168_20 B, 28.04.2020) prior to the beginning of the study. The trial was performed in accordance to the declaration of Helsinki. Patients were recruited following predefined inclusion criteria: I) Neonates with non-syndromic orofacial cleft with their first consultation at the Department of Orthodontics and Orofacial Orthopedics within the first days and weeks of life, II) neonates without orofacial cleft (born in the Department of Gynecology and Obstetrics Erlangen with ongoing regular consultations in local pediatric practices) and III) written informed consent by the parents and/or legal guardians. Exclusion criteria were defined as the following: I) Neonates with syndromic cleft lip and palate, II) preterm birth (< 37 weeks gestational age), III) neonates with underweight at birth (<2500g), IV) neonates with systemic and metabolic or autoimmune diseases, V) neonates with antibiotic intake, VI) revoked written informed consent by the parents and/or legal guardians. Two informed consent forms for participation in the trial and for utilization of tongue smear samples, data protection sheets and information material explaining the study in adequate language were provided. Written informed consent forms and data protection sheets by the parents and/or legal guardians were mandatory for enrollment in the trial. Moreover, written questionnaires were given to the parents and/or legal guardian to collect information about neonates’ clinical parameters including weight and height at birth, nutrition protocol, intake of antibiotics and/or supplements as well as to collect information about the mother including information type of birth and intake of antibiotics intrapartum (**Table 2**). Neonates were included regardless of birth type (vaginal birth or *via* C-section) and, hence, neonates whose mothers received intrapartum antibiotics due to C-section, were not excluded.

**Table 2:** Characteristics of Study population.

**Characteristics of the control group**									
#	Age at T0 (d)	Age at T1 (d)	Gender	Weight T0 (g)	Height T0 (cm)	Type of birth^1^	PROM^*^	Antibiotics^2^	Nutrition^3^
001	2	36	f	3350	52	1	0	2	0
002	2	34	m	3800	53	1	0	2	0
003	2	27	m	4120	55	1	0	2	0
004	2	23	f	3150	50	1	1	2	0
005	2	32	m	3340	50	0	0	0	0
008	3	29	f	3180	51	0	1	0	0
009	2	35	m	4030	54	0	0	0	0
010	2	38	m	3640	53	0	0	0	0
011	2	31	m	3050	50	0	1	0	0
012	2	20	f	2930	50	0	1	0	0
013	3	32	m	3670	54	0	1	0	0
015	3	34	f	3200	50	0	1	0	0
016	2	42	m	3940	53	1	1	2	0
018	2	24	f	3570	54	0	0	0	0
020	3	36	f	4200	56	1	0	2	0
021	3	22	m	3480	54	1	0	2	0
022	3	24	m	2950	50	0	0	0	0
**Characteristics of the study group (CLP)**									
#	Age at T0 (d)	Age at T1 (d)	Gender	Weight T0 (g)	Height T0 (cm)	Type of birth^1^	PROM^*^	Anti-biotics^2^	Nutrition^3^
001	7	24	m	2590	48	0	0	2	2
002	n.d.	19	m	3130	44	1	0	1	2,*4*
003	3	38	m	3040	51	1	0	1	2
004	5	n.d.	f	2980	51	0	0	0	0
005	2	37	f	2940	51	0	0	0	1
007	2	29	m	3220	51	0	0	0	1
009	3	29	m	3240	49	0	0	1	3
010	3	38	m	3320	51	1	0	1	3,*4*
011	3	34	m	3350	53	0	0	0	1
012	3	25	m	3900	51	0	0	0	2,*4*
014	2	31	m	3120	47	1	0	1	2
015	1	22	f	2800	51	0	0	0	2
016	11	39	f	3120	51	0	0	0	1,*4*
017	14	40	m	2860	50	0	0	0	3
018	8	34	f	3890	51	0	0	0	0
**Special characteristics of the study group: classification, severity, type of treatment**									
#	Etiology^4^	BCLP*	UCLP*	CPo*	CLo*	LAHSHAL Code^5^	LAHSHAL Severity^6^	Severity Score^7^	pAM*
001	ps	0	1	0	0	- - - SHAL	0002222	8	1
002	s	0	0	1	0	- - hSh - -	0012100	4	0
003	ps	0	1	0	0	- - - SHAL	0002222	8	1
004	s	0	0	1	0	- - hSh - -	0012100	4	0
005	s	0	0	1	0	- - HSH - -	0022200	6	1
007	ps	0	1	0	0	- - - SHAl	0002221	7	1
009	ps	1	0	0	0	lAHS - - l	1222001	8	1
010	ps	1	0	0	0	LAHSHAL	2222222	14	1
011	ps	0	1	0	0	LAHS - - -	2222000	8	1
012	ps	1	0	0	0	LAHSHAL	2222222	14	1
014	ps	0	1	0	0	lAHS - - -	1222000	7	1
015	ps	1	0	0	0	laHSHAL	1122222	12	1
016	s	0	0	1	0	- - HSH - -	0022200	6	1
017	s	0	0	1	0	- - HSH - -	0022200	6	1
018	p	0	0	0	1	la - - - - -	1100000	2	0

s, cleft of the secondary palate; p, cleft of the primary palate; ps, cleft of the primary and secondary palate; d, days; g, grams; cm, centimeter; f, female; m, male; n.d., not done

* 0, no; 1, yes; ^1^ v, vaginal; c, caesarian.

^2^ 0 = no antibiotic intake of neonates or mother before birth, 1 = mother before birth, 2 = neonates after birth.

^3^ 0 = breastfeeding, 1 = bottle feeding breast milk, 2 = bottle feeding partly breast milk, partly artificial formula, 3 = bottle feeding artificial formula, 4 = postnatal tube feeding for <1 week (=T0).

^4^ p = cleft of the primary palate, s = cleft of the secondary palate, c = combined clefting of the primary and secondary palate.

^5^ LAHSHAL Code: minus sign (-) =not affected, small letter = incompletely affected, capital letter = completely affected.

^6^ LAHSHAL Severity: 0 = not affected, 1 = incompletely affected, 2 = completely affected.

^7^ LAHSHAL Score, sum of the LAHSHAL Severity.

### 2.2 Recruitment

**Figure 1:**
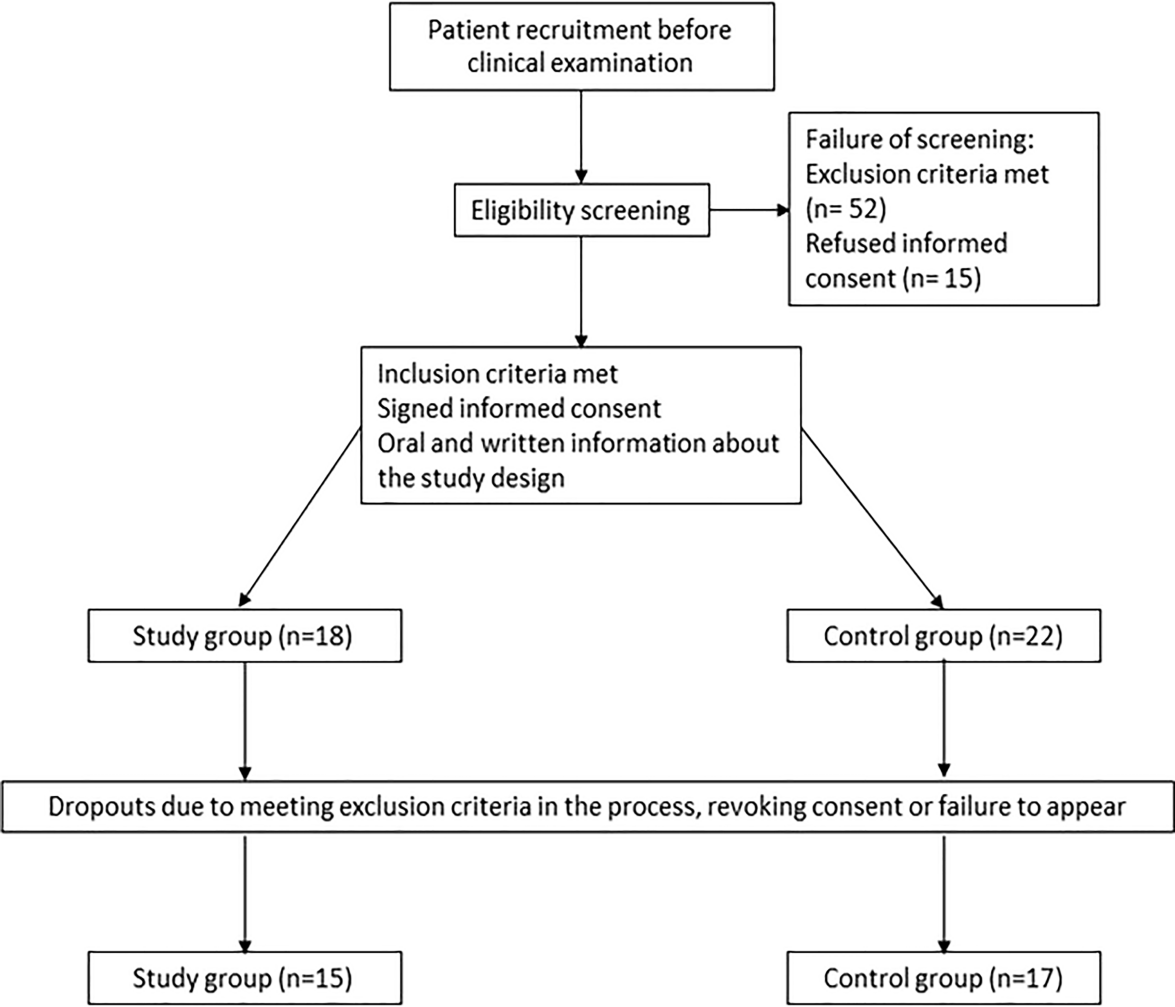
Flow of patients.

After eligibility screening, a total of 40 study participants were enrolled in this study and diveded into two groups (**Figure 1**). The study group, neonates (n=18) with orofacial clefts, was recruited at the Department of Orthodontics and Orofacial Orthopedics, Universitätsklinikum Erlangen, Friedrich-Alexander Universität (FAU) Erlangen-Nürnberg. The control group, neonates without orofacial clefts (n=22), was recruited at the Department of Gynaecology and Obstetrics, Universitätsklinikum Erlangen, FAU Erlangen-Nürnberg. Dropouts were registered due to the following reasons: 1) Failure to appear to the consultation and study appointments (n=1), 2) revoke of consent by the parents and/or legal guardians (n=4) or 3) meeting the exclusion criteria during the course of the study (n=3) (e.g. diagnosis of a syndrome or acute systemic or metabolic disease). In total, the dropout rate was 15% (n=8 in total, n=3 CLP patients and n=5 controls) with a final sample size of 15 study participants in the CLP group and 17 study participants in the control group (**Figure 1**).

### 2.3 Sample collection

Overall, 132 tongue smear samples were collected during a timespan ranging from June 2020 to June 2021. Tongue smear were obtained using sterile swabs and wiping carefully over the middle and anterior part of the tongue several times for about 10 seconds. Parents were instructed to pause feeding their infants 2-3 hours prior to sample collection and inform the study leader, if medical treatment (e.g., antibiotic treatment) was performed prior to sample collection or during the study course leading to an exclusion of the study participant. For the control group, sample collection was performed during routine appointments at the Department of Gynaecology and Obstetrics, Universitätsklinikum Erlangen, FAU Erlangen-Nürnberg (U2 = T0) as well as at local pediatricians: (U3 = T1). For the OFC group, the sample collection was performed at the Department of Orthodontics and Orofacial Orthopedics during initial consultation (T0) and during regular appointments (T1). For sufficient preservation, samples were stored on dry ice within seconds after sample collection performed on neonates and then either immediately frozen at -80° C or stored in freezers at -20°C for a maximum of 5 days and subsequently transferred to a ultra-low freezing unit at -80°C for definite storage. An uninterrupted cold chain was preserved permanently. Storage as well as further processing was conducted in the research laboratory of the Department of Orthodontics and Orofacial Orthopedics, Universitätsklinikum Erlangen, FAU Erlangen-Nürnberg.

### 2.4 Study population

Male and female subjects were distributed equally in both groups (**Tables 2**, **3**). The average age at T0 is slightly different between the OFC and the control group since initial clinical surveillance at orthodontists for treatment of OFC neonates is often slightly later than the initial consultation (U2) at gynaecologists (median age OFC neonates = 3 days; control neonates = 2 days) (**Tables 2**, **3**). Samples at T1 were collected at a median age of 32 days (CLP group) and 31 days (control group), hence, there were no differences in the distribution between the two groups (**Tables 2**, **3**). With a median weight of 3480 g, the control group was consistent with the European average 26, whereas orofacial cleft patients’ weight was significantly lower with a median weight of 3120 g (**Table 3**) which is in line with previous studies presenting evidence for reduced birth weight and height at birth 27 and belated growth and development mostly due to feeding difficulties 28-30. Similarly, there is a mild almost significant difference regarding the height at birth in both groups that is in average 51 cm for orofacial cleft patients and 53 cm for the control group (**Table 3**). The mode of birth (vaginal and caesarean) and antibiotic intake by mothers intrapartum were equally distributed in both groups (**Table 3**). However, in contrast to the mainly breast-fed control group, neonates with OFC were mostly fed with bottles (breast milk, mixed nutrition, exclusively formula-fed) and some required tube feeding after birth, which was expected due to the explained feeding issues in neonates with OFCs 14-17. The LAHSHAL classification scheme by Kriens et al. 10 uses letters to describe the cleft phenotype. Based on the LAHSHAL code 10, we created a severity score for subgroup investigations. Capital letters representing complete affection of the anatomical structure 10 were given by the value two (2), while small letters representing incomplete affection 10 were given the value one (1) and minus signs representing not-affected parts 10 were given the value zero (0). At the end, all numbers were summed up for each individual patient and the final sum value was used as severity score (the higher the final number, the more severe was the clefting) (**Table 2**).

**Table 3:** Descriptive statistics and statistical comparisons between CLP group and Control group.

	CLP group (n=15)	Ctrl group (n=17)		p-value
Age in days, median (IQR):				
At T0	3 (2-7.25)		2 (2-3)	**0.046** ^a^
At T1	32 (24.75-38)		31 (24-35.5)	0.717^a^
Gender:				
Female, n (%)	5 (33)		7 (41)	0.789^b^
Male, n (%)	10 (67)		10 (59)	0.561^b^
Birth weight (grams) median (IQR):	3120 (2920-3328)		3480 (3165-3870)	**0.024** ^a^
Birth height (cm) median (IQR):	51 (49.75-51)		53 (50-54)	**0.051** ^a^
Mode of Delivery:				
Vaginal, n (%)	11 (73)		10 (59)	0.659^b^
Caesarean section, n (%)	4 (27)		7 (41)	0.485^b^
Antibiotics:				
None, n (%)	9 (47)		10 (59)	0.638^b^
Mother before birth, n (%)	5 (33)		7 (41)	0.718^b^
Neonate after birth, n (%)	1 (7)		0 (0)	0.287^b^
Nutrition:				
Breastfeeding, n (%)	2 (13)		17 (100)	**0.001** ^b^
Bottle feeding breast milk, n (%)	3 (20)		0	0.065^b^
Bottle feeding partly breast milk, partly artificial baby food, n (%)	4 (27)		0	**0.033** ^b^
Bottle feeding artificial baby food	2 (13)		0	0.132^b^
Postnatal tube feeding for <1week=T0, n (%)	4 (27)		0	**0.033** ^b^

^a^Mann-Whitney Test; ^b^chi-square Test. Bold font = p ≤ 0.05.

### 2.5 Cytokine analysis

For the measurement of cytokine concentrations, collected tongue smear samples were isolated from swabs by centrifugation at 21.130 × g (1 minute at 4°C) as previously described by Seidel CL et al. (25). Then the samples’ volume was measured and diluted with diluent 43 (Mesoscale Discovery, R50AG-2) to a volume of >50 µL. A few samples needed to be diluted more than 14-fold, due to their very small initial volume (≤ 4µl). For some of these high diluted samples the cytokine-measurement failed because they resulted below the detection range. These not reliable concentration-values were filtered and excluded from the analysis. In particular, regarding the CLP group at T0, sample 018 was diluted 37-fold and excluded from GM-CSF, IL-10 and IL-4 analysis; sample 015 was diluted 28 times and excluded from IL-4 measurements. In the control group at T0 the sample 008 was diluted 37-fold and excluded from IL-4 measurements. In the control group at T1 the samples 009 and 010, diluted 22-fold, were included only for IL-8 and IL-1ß analysis, 003 was excluded from IL-4, IL-10 and TNF measurement. Cytokine concentrations were analyzed by multiplex immunoassay in 96 well plates with a U-PLEX Biomarker Group 1 (hu) assay (Mesoscale discovery; K15067L-2) on a MESO QuickPlex SQ 120 instrument (Mesoscale discovery). The assay was performed according to the manufacturer’s instructions. The elaboration of the data was made with the Program Mesoscale Discovery Workbench.

### 2.6 Statistics

The calculation of cytokine concentrations and statistical analyses was done with Microsoft Excel 2016 (Microsoft, Redmond, WA, USA) and GraphPad Prism 9 statistical software (GraphPad Software, San Diego, CA, USA). The data sets were analyzed by using the Mann-Whitney U-test, Chi Square test, Kruskal–Wallis test and Dunn’s multiple comparisons test. Differences were considered significant with p-values ≤ 0.05. Correlations between cytokines and severity-score were calculated with Pearson’s correlation coefficient.

## 3 Results

### 3.1 CLP neonates present significantly higher levels of pro-inflammatory mediators compared to controls

**Figure 2:**
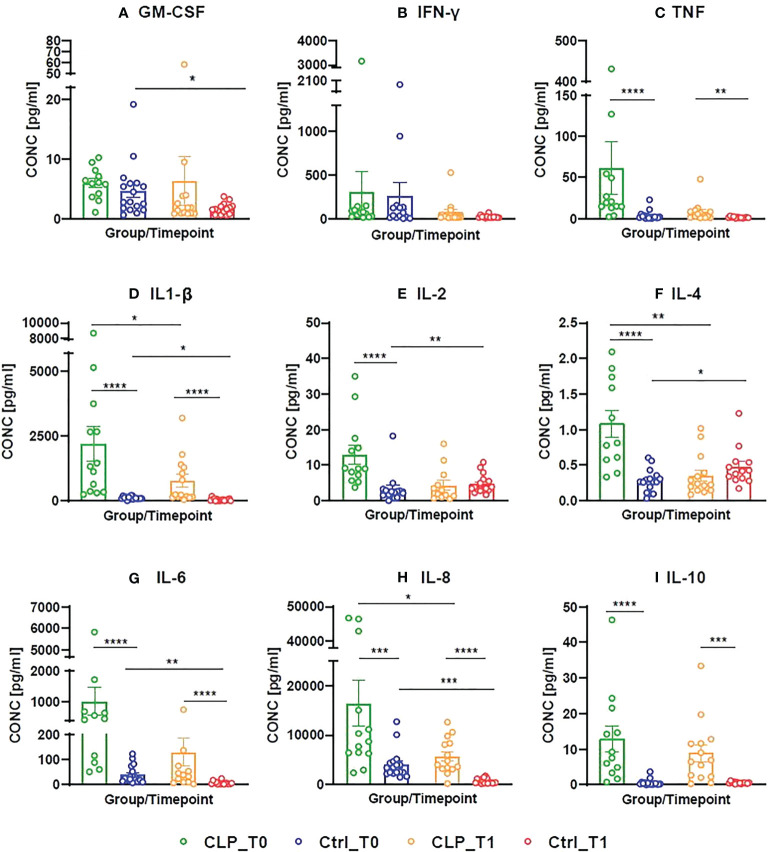
Concentrations (pg/ml) of measured cytokines (Granulocyte-macrophage colony-stimulating factor = GM-CSF, Interferon gamma = INF-y, Tumor-necrosis-factor = TNF, Interleukin (IL)-1ß/-2/-4/-6/-8/-10) in cleft patients (CLP) compared to controls (Ctrl) at both time points (T0 = after birth, T1 = 4-5 weeks after birth). A color scheme represents each group – time point – combination (CLP-T0: = green, Ctrl-T0 = blue, CLP-T1 = orange, Ctrl T1 = red). Histograms presenting the cytokine concentrations are given for each measured cytokine **(A-I)**, both groups (CLP vs. ctrl) and both time points (T0 vs. T1). Each histogram is a scatter dot plot with the mean and standard error of mean (SEM). The cytokine concentration in pg/ml is given on the Y axis, while the X axis represents the four groups: CLP_T0, Ctrl_T0, CLP_T1, Ctrl_T1. The comparisons were statistically analyzed with t test and Mann-Whitney U-test, statistically significant comparisons are indicated by *p ≤ 0.05, **p ≤ 0.01, ***p ≤ 0.001, **** p ≤ 0.0001.

The concentration of nine inflammatory mediators (GM-CSF, INF-γ, TNF, IL-1β/-2/-4/-6/-8/-10) in tongue smear samples was measured with multiplex immunoassay in each of the following four groups (**Figures 2A–H**): CLP patients (CLP) and healthy controls (ctrl) at T0 and T1. TNF, IL1-β, IL-6, IL-8 and IL-10 concentration were significantly higher in the CLP group compared to the control group at both time points (**Figures 2C, D, G–I**). The concentration of IL-2 and IL-4 was significantly higher in the CLP group, however only at T0 (**Figures 2E, F**). Considering the CLP group, the concentration of the pro-inflammatory cytokines IL-1β and IL-8 and the anti-inflammatory cytokine IL-4 decreased significantly from T0 to T1 (**Figures 2D, F, H**). Regarding the control group, the concentrations of pro-inflammatory cytokines (GM-CSF, IL-1β, IL-6 and IL-8) declined significantly from T0 to T1 (**Figures 2A, D, G, H**), while anti-inflammatory cytokines (IL-2 and IL-4) increased significantly from T0 to T1 (**Figures 2E, F**) resulting in higher levels of IL-4 at T1 in the control group than in the CLP group (**Figure 2F**).

To conclude, a general reduction of cytokine levels was observed in tongue smear samples within the first weeks of life in neonates. Moreover, several pro-inflammatory cytokines showed significantly higher concentrations in the CLP group compared to controls, while controls were characterized by highest levels of anti-inflammatory IL-4 after the first weeks of life.

### 3.2 Defining a numerical classification scheme to differentiate CLP neonates into low and high cleft severity

**Figure 3:**
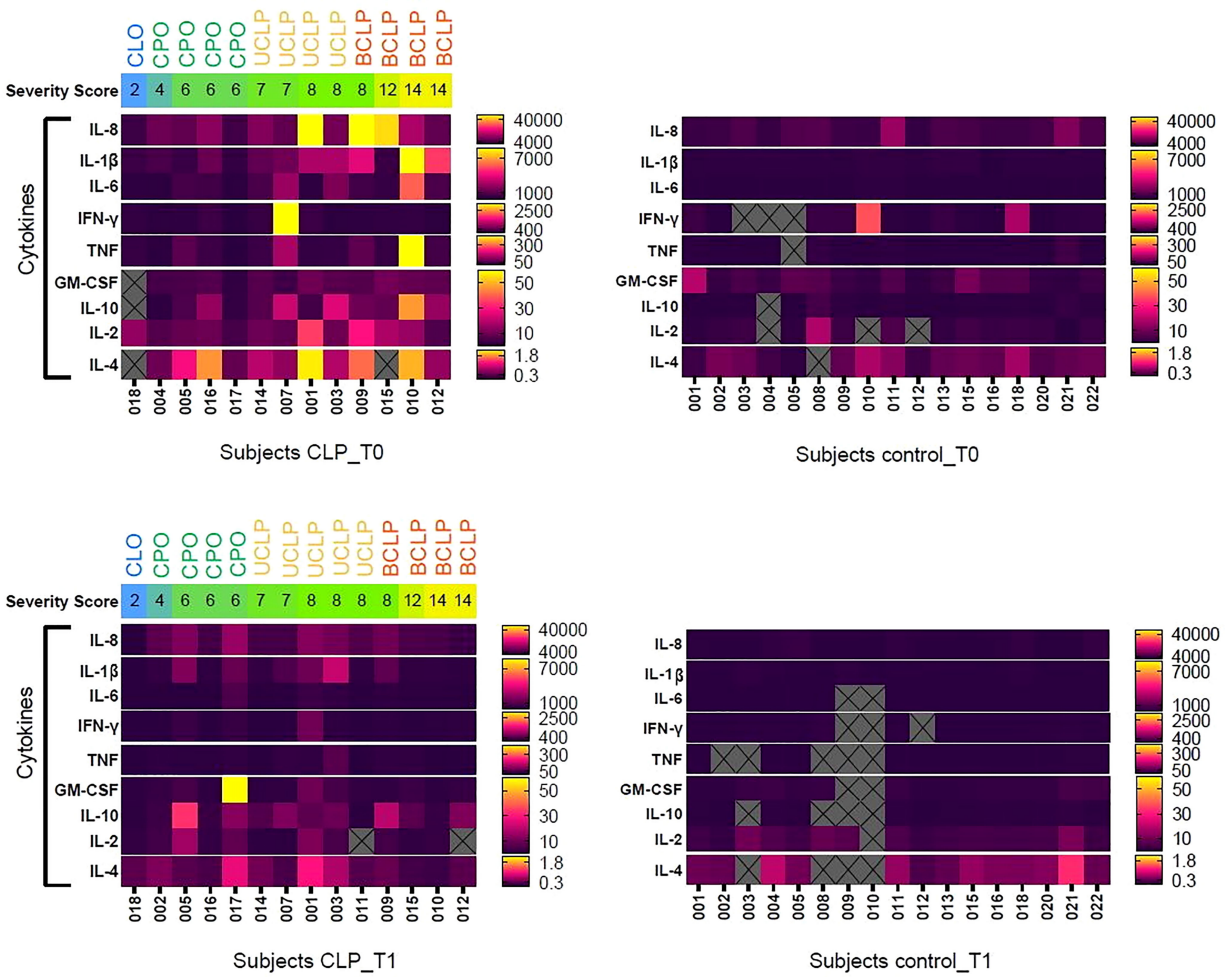
Heat map representing the cytokine concentrations (pg/ml) of measured cytokines (Granulocyte-macrophage colony-stimulating factor = GM-CSF, Interferon gamma = INF-y, Tumor-necrosis-factor = TNF, Interleukin (IL)-1ß/-2/-4/-6/-8/-10) for each subject in the cleft patients (CLP) group compared to the control (Ctrl) group at both time points (T0 = after birth, T1 = 4-5 weeks after birth). The subjects in the CLP group are organized left to right from the lowest severity score (severity score 2) to the higher (severity score 14). The severity score and the cleft type for each individual subject are ordered horizontally for each patient and are represented by color scheme: severity score is given by a color gradient from low (blue) to medium (green) to highest (yellow) cleft severity; cleft phenotype is given above: Cleft lip only (CLo) =blue letters, Cleft palate only (CPo) = green letters, unilateral cleft lip palate (UCLP) = yellow letters, bilateral cleft lip palate (BCLP) = orange letters. The red line represents the cut-off between low and high severity score. The range of concentration for each cytokine (pg/ml) is given on the right side and the measured cytokines are organized vertically from highest to lowest concentration. A double gradient map represents the levels of cytokines for each subject (yellow = highest concentration, pink = intermediate concentration, dark violet = lowest concentration). The gray squares with X are excluded multiplex-cytokine measurements due to concentrations below the detection range.

In order to investigate the impact of cleft severity numerically, we created a severity score according to the LAHSHAL classification 10 (**Table 2**, **Supplementary Figure 1**). The higher the sum of the LAHSHAL code, the more anatomical parts were completely affected by clefting, while low values indicate that solely the lip or the palate were affected by clefting (**Table 2**, **Supplementary Figure 1**). A heatmap analyses in accordance to severity score was performed (**Figure 3**). Interestingly, a trend of higher cytokine concentrations was seen in tongue smear samples from CLP neonates with higher severity score in comparison to CLP neonates with lower severity score at T0 (**Figure 3**) depicting a visual separation into CLP neonates with severity scores greater than value 7 and neonates with severity scores up to the value 7 (**Figure 3** red line). In accordance to the results found in the heat map, we defined a cut-off value dividing CLP patients in high cleft severity (severity score 8-14) and low cleft severity (severity score 2-7) for further investigations.

### 3.3 Neonates with high cleft severity present significantly higher levels of pro-inflammatory mediators compared to low cleft severity after birth

**Figure 4:**
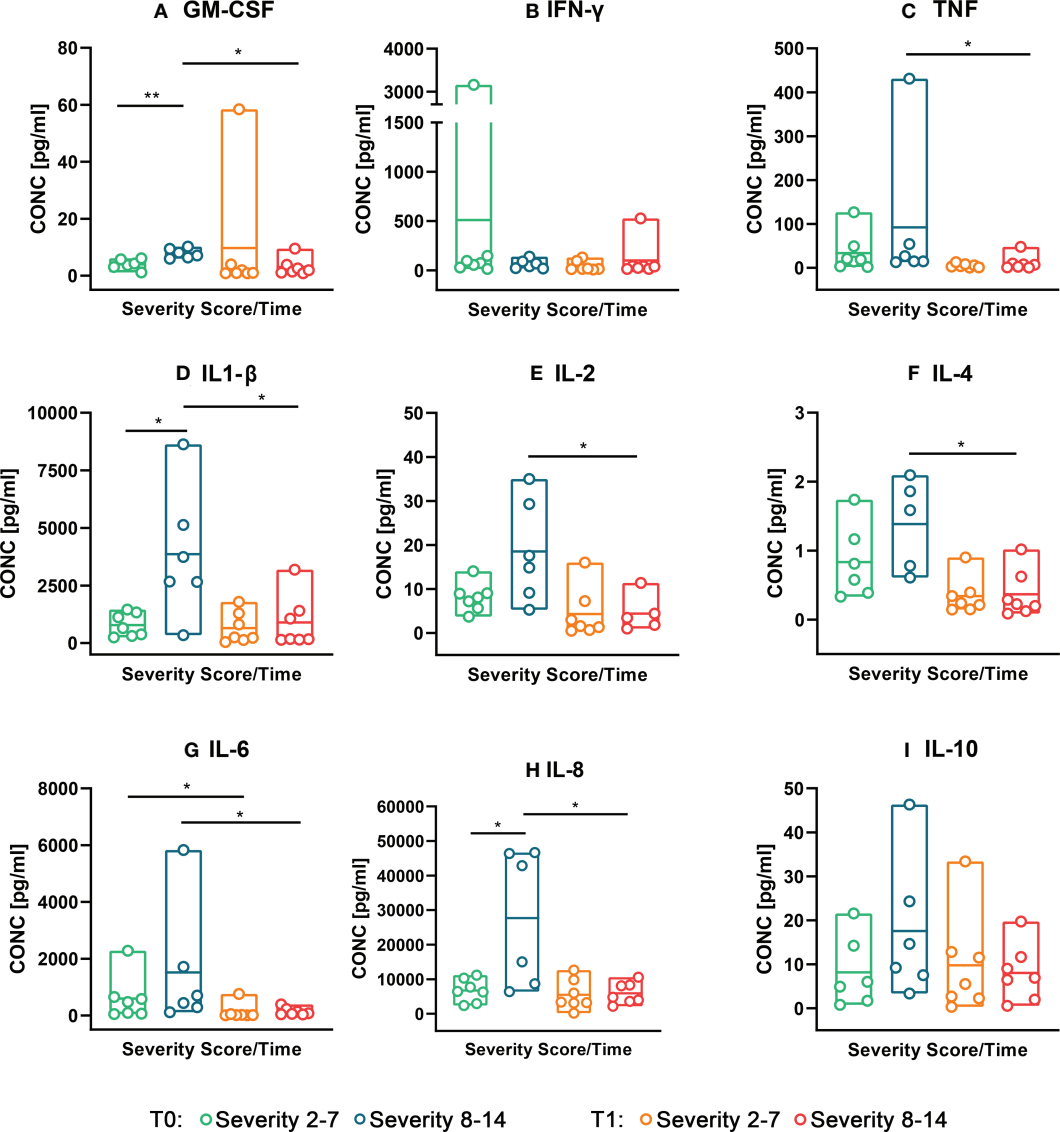
Concentrations (pg/ml) of measured cytokines (Granulocyte-macrophage colony-stimulating factor = GM-CSF, Interferon gamma = INF-y, Tumor-necrosis-factor = TNF, Interleukin (IL)-1ß/-2/-4/-6/-8/-10) in neonates with low cleft severity (Severity 2-7) compared to neonates with high cleft severity (Severity 8-14) at both time points (T0 = after birth, T1 = 4-5 weeks after birth). A colour scheme represents each group – time point – combination (Severity 2-7 T0: = green, Severity 8-14 T0 = blue, Severity 2-7 T1 = orange, Severity 8-14 T1 = red). The concentrations (pg/ml) of the measured cytokines (GM-CSF, INF-y, TNF, IL-1ß/-2/-4/-6/-8/-10) are given from the top left to the bottom right histogram **(A-I)**. Floating bars (max to min) with dots represent the cytokine concentration distributed in two subsets (Severity 2-7 and Severity 8-14) and both time points (T0 vs. T1). The horizontal line in the bars represents the mean. The statistical analysis was made with Mann-Whitney U-Test, *p value ≤ 0.05, **p ≤ 0.01.

Interestingly, significantly higher pro-inflammatory cytokine concentrations (GM-CSF, IL-1ß and IL-8) were seen in tongue smear samples from neonates with high cleft severity compared to low severity at T0 (**Figures 4A, D, H**). All other measured cytokines (except for IFN-γ) also presented elevated levels in the high severity group compared to the low severity group (**Figure 4**). Moreover, in CLP neonates with high cleft severity, most measured cytokines (TNF, IL-1β/-2/-4/-6/-8, GM-CSF) showed a significant reduction from T0 to T1 (**Figures 4A, C–H**), while in neonates with low severity solely IL-6 decreased significantly (**Figure 4G**).

### 3.4 Distinct cytokine correlation clusters were found in each group for each time point

**Figure 5:**
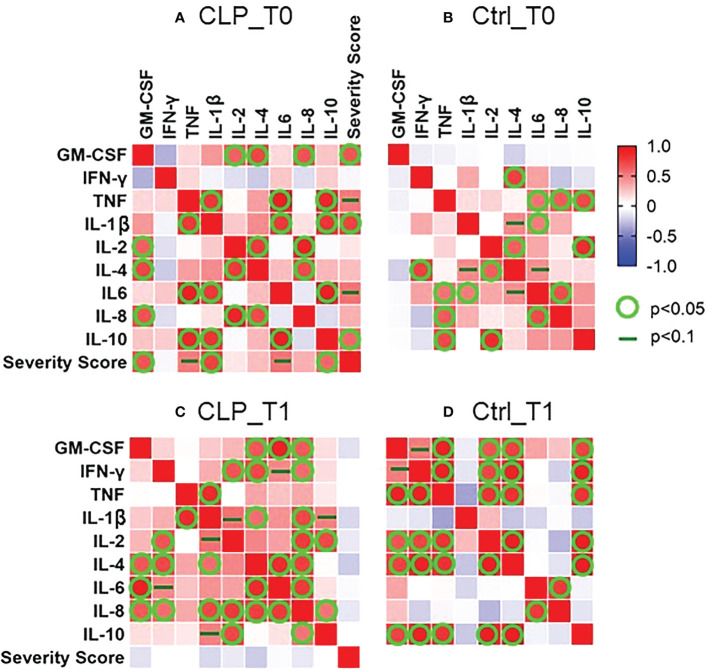
Pearson correlation matrix between concentrations (pg/ml) of measured cytokines (Granulocyte-macrophage colony-stimulating factor = GM-CSF, Interferon gamma = INF-y, Tumor-necrosis-factor = TNF, Interkeukin-1ß/-2/-4/-6/-8/-10) and severity score for cleft patients (CLP) compared to controls (Ctrl) at both time points (T0 = after birth, T1 = 4-5 weeks after birth) The heat maps represent the correlation matrix for the CLP group at T0 **(A)** and T1 **(C)** and the controls at T0 **(B)** and T1 **(D)**. The correlations were calculated between the measured cytokines (GM-CSF, INF-y, TNF, IL-1ß/-2/-4/-6/-8/-10) and the severity score. A color scheme represents the Pearson’s r for each combination of variables. Red presents a perfect positive correlation (r = 1) and blue a perfect inverse correlation (r = -1). No correlation (r = 0) is represented with white color. A statistically significant correlation (p<0.05) is represented by a green circle and a dark green line indicates a tendentially significant correlation (p<0.1).

To investigate the relationship between cytokine concentrations in tongue smear samples and orofacial cleft severity, Pearson correlation analysis was performed for both groups (CLP, ctrl) and both time points (T0, T1). Regarding the CLP group at T0, positive correlations were seen for: 1) GM-CSF, IL-2/-4/-8; 2) TNF, IL-1β/-6/-10 (**Figure 5A**). Considering the control group at T0, the following positive correlations were detected: 1) TNF, IL-6/-8/-10; 2) IL-2/-4/-10; 3) IL-4, IFN-γ; 4) IL-1β/-6 (**Figure 5** B). As for the CLP group at T1, IL-8 correlated positively with all measured cytokines (GM-CSF, IFN-γ, IL-1β, -2, -4, -6, -10) except for TNF (**Figure 5C**) and IL-4 correlated with all measured cytokines (GM-CSF, IFN-γ, IL-1β, -4, -6) except for TNF, IL-2 and IL-10 (**Figure 5C**). Furthermore, correlations were seen for: 1) TNF, IL-1ß; 2) IL-2/-10 (**Figure 5C**). Considering the control group at T1, positive correlations were seen for GM-CSF, TNF, IL-2/-4/-10 (**Figure 5D**), while the pro-inflammatory mediators IL-1β/-6/-8 correlated negatively with all measured cytokines (**Figure 5D**). Considering the severity score in the CLP group, positive correlations with pro-inflammatory mediators (GM-CSF, IL-1β) were seen at T0 (**Figure 4A**).

### 3.5 Breast-milk (bottle) feeding correlated with reduced levels of pro-inflammatory cytokines IL-6 and IL-8 in neonates with orofacial clefts

In contrast to control neonates, most neonates with CLP suffer from feeding difficulties and require bottle feeding and in very severe cases even postnatal tube feeding after birth. Overall, a trend to higher cytokine levels in tongue smear samples was seen in bottle-fed neonates receiving mixed baby food (MF) and artificial food (AF) compared to the breast-milk (bottle) fed group (BM) at T0, while differences were only significant for IL-8 (**Supplementary Figure 3**). At T1, IL-6 displayed significantly higher levels in tongue smear samples from neonates receiving artificial baby food compared to breast-milk (bottle) feeding (**Supplementary Figure 3**). Notably, one neonate (LKG_017) with CPo presented a peak of GM-CSF at T1 (**Figure 3**), which received AF exclusively (**Table 2**).

## 4 Discussion

OFCs present different phenotypes and severities (10, 11) and are characterized by an insufficient separation of oral and nasal cavity (10, 11) hereby presenting a risk for intraoral inflammation (20). However, characterisation of local oral cytokine milieu in non-invasively collected tongue smear samples has neither been performed in healthy neonates nor in neonates with OFC. In order to identify inflammatory alterations and potential risk factors for wound healing disorders, we investigated cytokine concentrations in tongue smear samples from neonates with OFC compared to controls and correlated them with cleft phenotypes and severity.

The non-invasive sampling method using tongue smear samples was chosen due to several reasons. So far, two invasive methods were used to analyse cytokine concentrations in infants with OFC: 1) Two previous studies (23, 24) collected lip tissue during cleft surgery in infants (3-18 months of age) to investigate cytokine concentrations, but lacked an adequate control group since surgical collection of lip tissue from healthy neonates would be ethically inacceptable. 2) One study investigated cytokine concentrations and osteocalcin in peripheral blood samples collected from children with OFC (0-12 months, 1-3 years, 4-9 years, 10-15 years; n=80) compared to an age-matched control group (n=10/per age group) (63). The authors detected significantly higher pro-inflammatory cytokine concentrations in OFC children and distinct age-related correlations between IL-4 and osteocalcin with a focus on immune-skeletal interactions and postnatal osteogenesis (63), however, they did not detect differences between different cleft phenotypes and did not correlate their results to oral parameters. With respect to non-invasive methods to investigate cytokine concentrations in the oral cavity, several methods were used in patients with OFC: 1) Collection of stimulated saliva (64) or unstimulated saliva (65) using the spitting method: patients are asked to collect saliva in their mouth in an upright position with the head slightly tilted forward (unstimulated saliva: without moving the head or regurgitation) and to spit the collected saliva in a sterile tube several times until the required amount of saliva is collected (65); 2) Usage of sterile swabs to collect smear of defined areas, e.g., wiping over the tongue or the palate several times (areas with low saliva flow rate) or wiping over the cheek or sublingual area (high saliva flow rate) (25); 3) Sampling of gingival crevicular fluid by putting sterile paper strips in gingival pockets for a defined amount of time (in dentulous individuals) (25, 66–70); 4) Gathering of dental biofilm [supragingival plaque (25) or subgingival plaque (71)] with sterile dental instruments (in dentulous individuals). Some authors used the term ‘saliva swab’ referring to non-invasive methods described above, e.g., the spitting methods (72, 73), or mixing up different methods, e.g., stimulation of saliva by coughing and wiping over tongue, cheek, palate and gums afterwards (74). A previous study detected distinct differences and similarities between the cytokine concentration in different oral niches and defined immunological metaniches (plaque and gingival crevicular fluid; tongue and hard palate; sublingual area and cheek) (25). We chose tongue smear samples as a representative of the metaniche ‘tongue and hard palate’, since the tooth-associated metaniche ‘plaque and gingival crevicular fluid is cannot be found in neonates and since metaniche ‘sublingual area and cheek’ was characterized by overall lowest cytokine concentrations due to the high saliva flow rate in this area (25). Moreover, neither the collection of unstimulated or stimulated saliva would be possible in neonates due to well-known cooperation difficulties in this age. The advantage of the collection of tongue smear samples is the applicability in neonates, the repeatability due to the defined methodology, the non-invasiveness and the local investigation of cytokine samples in the oral cavity.

As to cytokine detection using tongue smear samples from neonates, all the analyzed cytokines (GM-CSF, INF-γ, TNF, IL-1β/-2/-4/-6/-8/-10) were detected presenting a broad spectrum of concentrations ranging from the highest values represented by IL-8 (10000-40000 pg/ml) to lowest values represented by IL-4 (0.3-0.9 pg/ml). Similarly, a previous study detected highest concentrations of IL-8 and lowest concentrations of IL-4 in tongue samples from young adults with periodontal health (25). Using a more invasive sampling method (collection of lip tissue during cleft surgery) and an elderly study population (4-13 months of age) without a control group, Pilmane et al. (23) found lower concentrations relatively highest concentrations of TNF (36.93 pg/mL) and low cytokine concentrations of all other measured cytokines (IL-2 1.58 pg/mL, IL-4 1.06 pg/mL, IL-6 0.59 pg/ml, IL-10 1.13 pg/mL, INF-γ 0.79 pg/mL, GM-CSF 0.70 pg/mL), which is partly in contrast to our results investigating tongue smear samples. Taken together, our non-invasive sampling method and our measurements were sensitive enough to analyze small sample volumes (with some limitations in volumes below 4 µl requiring high dilution, mostly IL-4 and IL-10) and were able to detect even higher concentrations of cytokines compared to invasive sampling methods (23).

The oral mucosa, including the tongue mucosa, constantly interacts with the external environment and plays a pivotal role in maintaining the tolerance with the local symbiotic bacteria on the one hand and as a defense against pathologic microbes on the other (75). Thereby, epithelial cells of the oral and tongue mucosa and tissue specific immune cells communicate *via* cytokines and soluble mediators to maintain the physiological oral homeostasis (75) (**Table 1**). Considering healthy neonates without OFC, we found a similar order of magnitude considering cytokine concentrations at T1 compared to detected cytokines in smear samples from the tongue of orally healthy adults (25): As to longitudinal changes of the cytokine levels in tongue smear samples, we found that the concentration of most measured cytokines decreased significantly from T0 to T1 in both the CLP and the control group. In a mice model, it was reported that the oral epithelium thickened gradually after birth due to keratinization and exhibited adult features within the first month after birth resulting in less permeability and less vulnerability to microbial infections and that saliva flow was upregulated (76, 77). Further, longitudinal changes in microbial alpha diversity [species richness or evenness (78, 79)] and beta diversity [variance in species composition (78, 79)] were observed in neonates within the first weeks of life (19). Notably, while we observed a decline of cytokine levels from T0 to T1 in both groups, alpha diversity increased significantly from T0 to T1 (19). Further, while we found that differences between the OFC and control group regarding cytokine levels were more significant at T0, the distinction between both groups became more evident at T1 regarding beta diversity (19). Hence, the observed reduction of cytokine concentrations in tongue smear samples might be due to a gradual epithelium remodeling process and increased saliva flow rate changes. Interestingly, the observed attenuated immunological reaction from T0 to T1 was contrariwise to reported increased microbial changes from T0 to T1 (19) indicating that postnatal immunological and microbial processes in the oral cavity do not always depend on each other.

Further, a significant increase of IL-2 and IL-4 from T0 to T1 was found resulting in highest levels of IL-4 at T1 in neonates without OFC. Regarding age-related changes of cytokine levels (in peripheral blood), children without OFC were characterized by a significant increase of IL-4 between 1 year and 3 years of age (63). IL-2 is an important regulator in communication of innate and adaptive immunity, e.g., by activation of T/B/NK cells (32); promotes tissue integrity, defense and tolerance in wound healing processes (50) and is a key regulator of regulation of oral mucosal inflammation (51). IL-4 is well-known for antagonistic effects in inflammatory diseases (inhibition of pro-inflammatory cytokines) (61) and enholds anti-inflammatory and immunoregulatory functions in mucosal immune reactivity (60) (**Table 1**). Further, IL-4 has a main role in wound healing by activation of fibroblasts, keratinocytes, neoangionesis and reepithelization (46) and, notably, high levels of IL-4 (or application of IL-4) were associated with accelerated mucosal wound healing (59, 62), hereby strengthening the wound (**Table 1**). Contemplating, the increase of IL-2 and IL-4 in healthy neonates might play an important role in maintaining oral mucosal homeostasis within the first weeks of life when neonates cope with an increase of microbiota in the oral cavity (19).

Regarding local cytokine concentrations in tongue smear samples from neonates with OFC, we found significantly higher levels of TNF, IL1-β, IL-6, IL-8 and IL-10 compared to controls at both time points. A study comparing lip tissue from infants with OFC (3-18 months) during cleft surgery compared to mucosal tissue gained during extraction therapy from adolescents with hyperdontia (non-age-matched control) showed a higher concentration of TNF in lip tissue from neonates with OFC compared to adolescent controls without OFC (24). Another study investigating lip tissue from infants with OFC (4-14 months) during cleft surgery without a control group, found overall highest concentrations of TNF compared to other measured cytokines in lip tissue from neonates with OFC (23). Considering systemic cytokine levels (in peripheral blood), infants with OFC (0-12 months) presented significantly increased levels of IL-17 and INF-γ compared to an age-matched control group, however, levels of IL-6 and IL-8 were similar in both groups (63), which is in contrast to our results found in tongue smear samples. TNF is a primary pro-inflammatory cytokine (37) and promotes vasodilatation, edema formation, leukocyte adhesion, regulation of blood coagulation (28). Remarkably, high levels of TNF were associated with surgical site infection (34) and worsening of the mucosal epithelial barrier function (40, 41), while TNF inhibitor treatment was shown to reduce surgical site infection (35, 36) (**Table 1**). IL1-β triggers primary pro-inflammatory reactions by the innate immune system, e.g., by activation of IL-8 (37), primary host defence responses to injury as well as enhancement of tissue damage in injury-associated mechanisms (44) (**Table 1**). IL-6 promotes migration of immune cells to damaged sites (45) and increased levels were shown to alter tissue integrity (41) (**Table 1**). IL-8 is a secondary pro-inflammatory cytokine in inflammatory reactions by the innate immune system stimulated by IL-1β and TNF (37, 38), induces tissue destruction by neutrophil accumulation and granules release (47, 48) and reduced IL-8 production was associated with almost scarless wound healing (49). IL-10 is commonly known as anti-inflammatory cytokine promoting oral tolerance (55), controlling the extend of inflammation in wound healing (52, 53) and down regulation of immune responses to pathogens/microbiota in mucosal inflammation (52), but it was also shown to have pro-inflammatory effects in a compromised immune-environment (54) and was shown to be upregulated during inflammatory processes and gingival inflammation when other pro-inflammatory cytokines increase, e.g. IL-2, INF-γ in gingival crevicular fluid (33, 56, 57) (**Table 1**). Therefore, higher levels of TNF and IL1-β/-6/-8 found in neonates with OFC might contribute to altered mucosal barrier and tissue integrity hereby increasing the risk for impaired wound healing. Notably, IL-10 seems to be upregulated next to other pro-inflammatory cytokines in the altered oral milieu of neonates with OFC.

Concerning different cleft phenotypes and severities, we detected higher levels of GM-CSF, TNF, IL-1β/-6/-8 in neonates with high cleft severity (complete UCLP/BCLP) compared to low cleft severity (CPo) at T0. Further, neonates with CPo presented significantly lower levels of IL-2 compared to UCLP/BCLP. Differences between different cleft phenotypes and severities diminished at T1 probably due to the significant reduction of cytokine levels in the BCLP group. Pilmane et al. (23) detected higher levels of IL-2, GM-CSF and TNF in lip tissue (collected during lip surgery) of neonates with UCLP/BCLP neonates compared to neonates with CPo, however, those differences were found at a later time point (4-18 months of age) and are not comparably to T0 or T1. As discussed above, both TNF, IL1-β, IL-6 and IL-8 levels were shown to compromise mucosal barrier function (40, 41) and high levels were associated with wound healing disorders (34, 44, 49) (**Table 1**). GM-CSF is key player in inflammatory signaling and dendritic cell recruitment into mucosa (29) and GM-CSF activated phagocytes cause tissue damage during wound healing (26). IL-2 is an important regulator in the communication of innate and adaptive immunity, holds both anti- and pro-inflammatory functions (32), regulates oral mucosal inflammation and increases migration of immune cells, fibroblasts and capillaries into damaged tissue (hereby strengthening the wound) (45). Interestingly, treatment with IL-2 was shown to promote tissue integrity, defense, tolerance and strengthens the wound (43, 45, 50) (**Table 1**). Hence, neonates with high severity score and with clefts affecting the lip and alveolus (BCLP/UCLP) presenting higher levels of GM-CSF, TNF, IL-1ß/-6/-8 on tongue smear samples might therefore be more at risk for progression of inflammatory processes than neonates with low severity score or cleft of the palate only (CPo). Whereas in CPo neonates, the lack of IL-2 might be associated to surgical site infection presenting a risk for residual clefts or fistulas in the hard palate. The higher cytokine concentrations found in UCLP/BCLP neonates compared to CPo neonates might be explicated by the affection of extraoral structures hereby leading to an altered microenvironment considering different aspects: 1) The affection of extraoral structures leads to an incompetent mouth closure and increases the airflow in the oral cavity. This might not only lead to altered immune reactions, but also to more ‘evaporation or dehydration’. A previous study investigating the saliva of 5-year-old children with OFC did not detect differences in saliva secretion rate (mL/min) between children with OFC compared to controls without OFC (80), however, so far no study evaluated saliva flow rate in newborns, which is probably due to the missing compliance for usual methods to measure saliva secretion rate (measuring the amount of time for collection of a defined amount of saliva using the spitting method). 2) Differences detected between CPo and UCLP/BCLP neonates regarding cytokine concentrations can be explained by altered oral microbiota in both phenotypes. Significant differences between neonates with high cleft severity (UCLP/BCLP) and low cleft severity (CPo) were found regarding beta diversity, which were more distinct at T1, and alpha diversity, presenting lowest alpha diversity in neonates with high cleft severity (UCLP/BCLP) at T0 (19). Since low alpha diversity is linked to higher inflammation levels, the low alpha diversity in UCLP/BCLP neonates might explain the higher concentrations of pro-inflammatory cytokines in neonates with UCLP/BCLP. Taken together, evaporation might play a role, but the high cytokine concentrations in UCLP/BCLP can also be elucidated by other factors, e.g., the interplay with oral microbiota.

Considering correlations between cytokines in neonates with OFC, we found strong positive correlations between 1) GM-CSF and IL-6/-8, 2) IL-1β and TNF and 3) IL-6/-10 and TNF at T0. Notably, the cleft severity score correlated positively also with GM-CSF, IL-1β and IL-10 at T0. During inflammatory reactions by the innate immune system, GM-CSF is capable to stimulate TNF production (28), while TNF and IL-1ß were shown to stimulate IL-8 production in mucosal cells (38, 39) and IL-6 is produced after IL-1 β, TNF and INF-γ stimulation (32) (**Table 1**). IL-10 was shown to inhibit the production of IL-1β and TNF (32), however, it holds also pro-inflammatory functions in a comprised micro-environment (54) (**Table 1**). Similar to a previous study presenting positive correlations between IFN-γ and IL-2 as well as IL-4 with IFN-γ in lip tissue of infants with OFC (23), we also detected positive correlations between IL-2 and INF-γ and between IL-4 and IFN-γ at T1 in OFC neonates. Control neonates were characterized by negative correlations between GM-CSF and all other cytokines at T0 and between IL-1β/-6/-8 with all other cytokines. During inflammation, a stimulation between GM-CSF and IL-1β/-6/-8 (28, 32, 38, 39) was observed (**Table 1**). Taken together, the cross-upregulation mechanisms between IL-1β/-6/-8 (and IL-10), TNF and GM-CSF might be linked to activation of inflammatory pathways in neonates with OFC shortly after birth, especially in neonates with high cleft severity, while in neonates without OFC a cross-regulation between those primary and secondary cytokines was not observed. Notably, while correlations between cytokine concentrations were mainly seen at T0, a previous study that microbial differences between neonates with OFC compared to controls were more distinct at T1 (19). Hence, we suppose that those cytokine interactions might be linked to prenatal or very early immunological reactions shortly after birth.

With regard to nutrition methods, the control group received breast-feeding only, while neonates with OFC presented individual nutrition modes due to feeding issues (14–17). Different nutrition methods were distributed equally with regard to cleft phenotype and severity (**Table 2**). Significantly higher pro-inflammatory cytokine levels (e.g., IL-6 and IL-8) were seen in bottle-fed neonates receiving mixed and completely artificial baby food compared to the breast-milk (bottle) fed group (data not shown). Breast milk encloses anti-inflammatory cytokines, e.g., TGF-β, IL-4/10, that can have an effect on oral tolerance and regulate immune responses (81). Hence, a positive impact of breast-milk (bottle) feeding on oral immunity can be supposed, however, larger sample sizes are needed in future studies and eventually further inflammatory mediators should be evaluated.

To conclude, this study showed that the sampling methodology using swabs is suitable for the detection of oral cytokine concentrations in neonates and presents a non-invasive alternative compared to tissue sampling. Further, early life physiological immune responses in the oral cavity seem to be characterized by high levels of oral inflammatory mediators after birth. Within the first weeks of life a significant decrease is detectable probably due to adaptation processes due to gradual epithelium remodeling. While a reduction of cytokine concentrations was found, a previous study detected an increase of microbial alpha and beta diversity was found in neonates within the first weeks of life (19). Hence, future studies should focus on the dissimilarities between the postnatal immunological and microbial reactions in the oral cavity within the first weeks of life and investigate whether possible prenatal immunological alterations might help to explain the presented high cytokine concentrations within the first days after birth. Interestingly, neonates without OFC were characterized by an elevation of IL-2 and IL-4 from T0 to T1 indicating that these patterns might be representable for physiological oral homeostasis (75) and ‘symbiosis’ (58, 82). Our results have high clinical relevance as we found that neonates with OFC (especially with high cleft severity) presented higher levels of pro-inflammatory cytokines. Further, pre-operative oral inflammation was associated with failure of intraoral surgeries (20), hence, the high inflammation found in neonates with OFC in early life might be a major risk factor for pre-operative inflammation prior to lip surgery with 6-7 months of age. Since prophylactic use of antibiotics prior to cleft lip surgery did not reduce the risk for wound healing disorders (21), we assume that preventive strategies to reduce pre-surgical inflammation should start after birth or at least as long as necessary to reduce the inflammatory state. Future studies should investigate oral cytokine concentrations using the non-invasive sampling method described here to investigate cytokine concentrations prior to and after surgical lip and/or palate closure to identify subjects with enhanced risk for wound healing disorders. Further, it would be interesting to analyse whether cytokine levels remain elevated after surgical lip and palate closure since a higher prevalence for gingivitis and periodontitis was found in children and adolescents with OFC (83, 84). In case that those studies would identify neonates at risk for wound healing disorders or an association with oral diseases later in life, new preventive strategies should investigate methods to reduce oral inflammation and to guide oral immune responses towards oral homeostasis. The encouragement of mothers of neonates with OFC to bottle feed their neonates with breast-milk rather than with artificial baby food might also be beneficial especially in severe cleft cases favoring an anti-inflammatory cytokine profile and the development of oral homeostasis.

## Data availability statement

The original contributions presented in the study are included in the article/**Supplementary Material**, further inquiries can be directed to the corresponding author.

## Ethics statement

This study has been approved by the local ethics committee of the Friedrich-Alexander-University Erlangen-Nürnberg (Krankenhausstraße 12, 91054 Erlangen, Vote number: 168_20 B, 28.04.2020) prior to the beginning of the study. Written informed consent to participate in this study was provided by the participants’ legal guardian/next of kin.

## Author contributions

Conceptualization, CS and LG; methodology, CS, EP, MatW; formal analysis, CS, MatW and EP; recruitment and sample collection CS, KS, MT and CU; clinical examination CS and KS; resources BF, UG and LG; cytokine analyses EP and CS; supervision of data analyses and definition of group/subgroup analyses CS; writing—original draft preparation CS and ES; review and critical discussion of cytokine-associated statements HS; writing—review MatW, IW, ManW, MS, BF, UG, MB. and LG; supervision LG; project administration CS and KS. All authors contributed to the article and approved the submitted version.

## Funding

This research funded by internal financial funding (ELAN) by the IZFK (Interdisziplinäres Zentrum für Klinische Forschung) by the Friedrich-Alexander Universität (FAU) Erlangen-Nürnberg (Grant holder: CS, Project number: P086).

## Acknowledgments

The authors thank all study participants and their parents and/or legal guardians for the participation in this study. A particular debt of gratitude is owed to Prof. Dr. M. Beckmann for the ability to recruit the control group in his Department as well as all doctors and nurses helping in the recruitment process. Special thanks should also be expressed to all the local pediatricians (Erlangen), who allowed, performed and helped with the sample collections during their routine investigations: PD. Dr. C. Plank; Prof. Koch and Dr. Gerdemann; Dr. Christian Döbig; Dr. Andrea Seiler, Dr. Jasmin Pletl-Maar and Dr. Gabriele Graf; Dr. Paul Wolf; Dr. Ulrike Lehnert; Dr. Karsten Naumann; Dr. Ulrike Scharnweber; Dr. Dorothea Schmitt-Colberg; Dr. Beate Kevekordes-Stade. Moreover, we thank Dr. Fabienna Mittermeier, Dr. Karoline März-Kalb as well as all doctors and nurses helping in the recruitment process in the Department of Orthodontics and Orofacial Orthopedics.

## Conflict of interest

The authors declare that the research was conducted in the absence of any commercial or financial relationships that could be construed as a potential conflict of interest.

## Publisher’s note

All claims expressed in this article are solely those of the authors and do not necessarily represent those of their affiliated organizations, or those of the publisher, the editors and the reviewers. Any product that may be evaluated in this article, or claim that may be made by its manufacturer, is not guaranteed or endorsed by the publisher.

## Supplementary material

Supplementary FIGURE 1 Schematic drawing of the LAHSHAL classification scheme and different cleft phenotypes is given: a) LAHSHAL scheme, b) Cleft Palate only (CPo), c) unilateral cleft lip and palate (UCLP) and b) bilateral cleft lip and palate (BCLP). The schematic drawing represents a occlusal view on the maxilla and a bottom view of the nose. The nose is presented on top of the scheme, followed by the lip, the vestibulum, the alveolus and the hard and soft palate and the pharynx on the bottom of the scheme. The parts affected by clefting are presented by discontinuities of the presented anatomical parts. The LAHSAL scheme uses letters to describe the affected anatomical parts (L = lip; A = alveolus, H = hard palate, S = soft palate).

Supplementary FIGURE 2 Concentrations (pg/ml) of measured cytokines (Granulocyte-macrophage colony-stimulating factor = GM-CSF, Interferon gamma = INF-y, Tumor-necrosis-factor = TNF, Interleukin (IL)-1ß/-2/-4/-6/-8/-10) in different cleft types (bilateral cleft lip palate = BCLP, unilateral cleft lip palate = UCLP, cleft palate only = CPo) at both time points (T0 = after birth, T1 = 4-5 weeks after birth). A color scheme represents each cleft phenotype – time point – combination (BCLP-T0: = blue, UCLP-T0 = dark green, CPo-T0 = light green, BCLP-T1 = red, UCLP T1 = orange, CPo T1 = bright orange). The concentrations (pg/ml) of the measured cytokines (GM-CSF, INF-y, TNF, IL-1ß/-2/-4/-6/-8/-10) are represented in this panel from the top left to the bottom right histogram (a to i). Floating bars (max to min) with dots (row data) represent the cytokine concentration distributed in each CLP subset (BCLP vs. UCLP vs. CPo; n = 4-5) at each time point (T0 vs. T1). The line in the bars represents the mean. The statistical analysis between the subsets at T0 and T1 was made with the Kruskal-Wallis test. The Mann-Whitney U-Test was applied for comparison between the two time points. Differences were considered significant with p-values ≤ 0.05.

Supplementary FIGURE 3 Concentrations (pg/ml) of measured cytokines (Granulocyte-macrophage colony-stimulating factor = GM-CSF, Interferon gamma = INF-y, Tumor-necrosis-factor = TNF, Interleukin (IL)-1ß/-2/-4/-6/-8/-10) in neonates with orofacial clefts at both time points (T0 = after birth, T1 = 4-5 weeks after birth) considering different nutrition modes (breast-milk (bottle) feeding (BM), mixed baby food (MF), artificial baby food (AF). A color scheme represents each group – time point – combination (BM-T0: = green, MF-T0 = light blue, AF-T0 = dark blue, BM-T1 = orange, MF T1 = rose, AF T1 = red). The concentration of the cytokines GM-CSF, INF-y, TNF, IL-1ß/-2/-4/-6/-8/-10 is represented in this panel from the top left graph to the bottom right. Each histogram, from top to the bottom, shows the concentration of a cytokine (pg/ml) in defined group-time point-combinations. Each histogram has floating bars (mean with max to min). The circles represent the row data. The statistical analysis was made with Kruskal-Wallis test (no statistical significance) and Mann-Whitney U-Test (dotted lines). Differences were considered significant with p-values ≤ 0.05.

## Glossary

**Table:**

A	Alveolous (LAHSHAL code)
BCLP	Bilateral cleft lip and cleft palate
C	caesarian
CCA	constrained correspondence analysis
CLo	Cleft lip only
CLP	Cleft lip and cleft palate
cm	centimeter
CPo	Cleft palate only
d	days
d.n.s.	data not shown
f	female
g	grams
GM-CSF	Granulocyte-macrophage colony-stimulating factor
H	Hard Palate (LAHSHAL code)
IL	Interkeukin
INF-γ	Interferon gamma
L	Lip (LAHSHAL code)
m	male
n	number
NAM	Nasoalveolar Molding
NGS	next generation sequencing
OFC	Orofacial clefts
p	Cleft of the primary palate
pAM	Passive Alveolar Molding
ps	cleft of the primary and secondary palate
s	Cleft of the secondary palate
S	Soft Palate (LAHSHAL code)
T	Tongue
T0	Time Point T0 after birth
T1	Time point T1 4-5 weeks after birth
TNF	tumor necrosis factor
UCLP	Unilateral cleft lip and cleft palate
v	vaginal
