# Supplementary figures and images for: Orofacial clefts lead to increased pro-inflammatory cytokine levels on neonatal oral mucosa
Source: Front Immunol. 2022 Nov 16;13:1044249. doi: 10.3389/fimmu.2022.1044249 (PMC9714580; doi:10.3389/fimmu.2022.1044249)

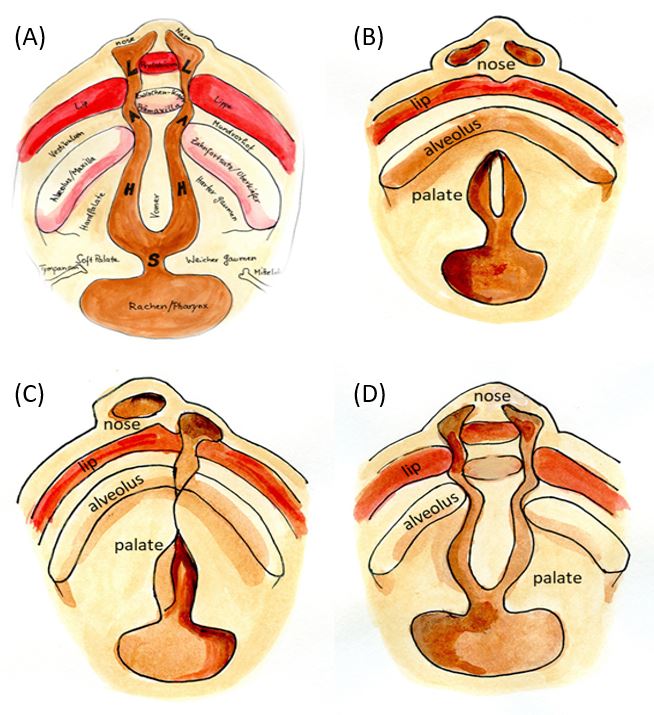

Supplement: Supplementary FIGURE 1 — Schematic drawing of the LAHSHAL classification scheme and different cleft phenotypes is given: a) LAHSHAL scheme, b) Cleft Palate only (CPo), c) unilateral cleft lip and palate (UCLP) and b) bilateral cleft lip and palate (BCLP). The schematic drawing represents a occlusal view on the maxilla and a bottom view of the nose. The nose is presented on top of the scheme, followed by the lip, the vestibulum, the alveolus and the hard and soft palate and the pharynx on the bottom of the scheme. The parts affected by clefting are presented by discontinuities of the presented anatomical parts. The LAHSAL scheme uses letters to describe the affected anatomical parts (L = lip; A = alveolus, H = hard palate, S = soft palate). [file Image_1.jpeg]

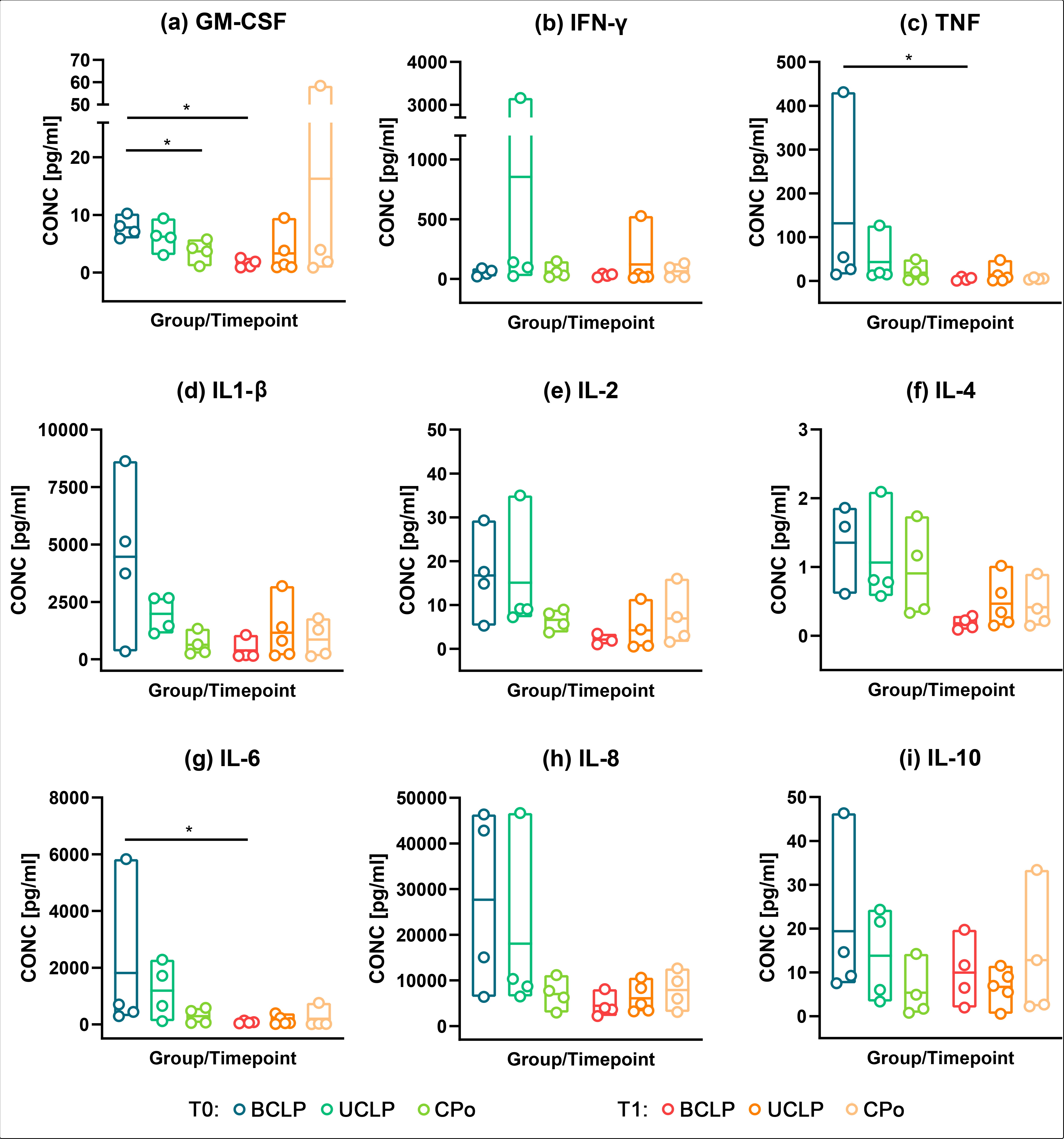

Supplement: Supplementary FIGURE 2 — Concentrations (pg/ml) of measured cytokines (Granulocyte-macrophage colony-stimulating factor = GM-CSF, Interferon gamma = INF-y, Tumor-necrosis-factor = TNF, Interleukin (IL)-1ß/-2/-4/-6/-8/-10) in different cleft types (bilateral cleft lip palate = BCLP, unilateral cleft lip palate = UCLP, cleft palate only = CPo) at both time points (T0 = after birth, T1 = 4-5 weeks after birth). A color scheme represents each cleft phenotype – time point – combination (BCLP-T0: = blue, UCLP-T0 = dark green, CPo-T0 = light green, BCLP-T1 = red, UCLP T1 = orange, CPo T1 = bright orange). The concentrations (pg/ml) of the measured cytokines (GM-CSF, INF-y, TNF, IL-1ß/-2/-4/-6/-8/-10) are represented in this panel from the top left to the bottom right histogram (a to i). Floating bars (max to min) with dots (row data) represent the cytokine concentration distributed in each CLP subset (BCLP vs. UCLP vs. CPo; n = 4-5) at each time point (T0 vs. T1). The line in the bars represents the mean. The statistical analysis between the subsets at T0 and T1 was made with the Kruskal-Wallis test. The Mann-Whitney U-Test was applied for comparison between the two time points. Differences were considered significant with p-values ≤ 0.05. [file Image_2.jpeg]

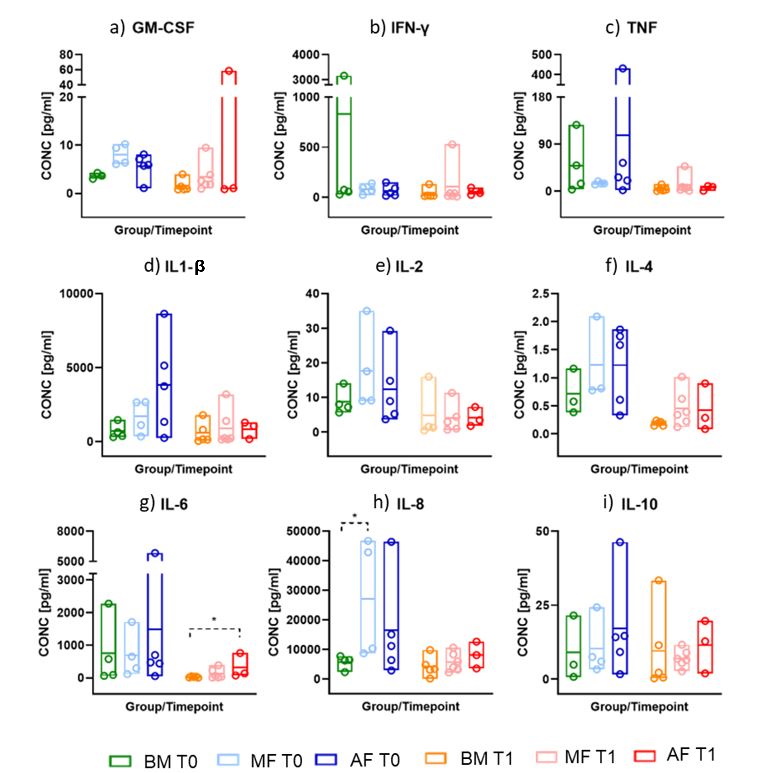

Supplement: Supplementary FIGURE 3 — Concentrations (pg/ml) of measured cytokines (Granulocyte-macrophage colony-stimulating factor = GM-CSF, Interferon gamma = INF-y, Tumor-necrosis-factor = TNF, Interleukin (IL)-1ß/-2/-4/-6/-8/-10) in neonates with orofacial clefts at both time points (T0 = after birth, T1 = 4-5 weeks after birth) considering different nutrition modes (breast-milk (bottle) feeding (BM), mixed baby food (MF), artificial baby food (AF). A color scheme represents each group – time point – combination (BM-T0: = green, MF-T0 = light blue, AF-T0 = dark blue, BM-T1 = orange, MF T1 = rose, AF T1 = red). The concentration of the cytokines GM-CSF, INF-y, TNF, IL-1ß/-2/-4/-6/-8/-10 is represented in this panel from the top left graph to the bottom right. Each histogram, from top to the bottom, shows the concentration of a cytokine (pg/ml) in defined group-time point-combinations. Each histogram has floating bars (mean with max to min). The circles represent the row data. The statistical analysis was made with Kruskal-Wallis test (no statistical significance) and Mann-Whitney U-Test (dotted lines). Differences were considered significant with p-values ≤ 0.05. [file Image_3.jpeg]
